# What Is an Identifier Good for? Issues in Using Visual Identifiers to Improve Care for People With Dementia in Hospital

**DOI:** 10.1111/jan.17039

**Published:** 2025-05-08

**Authors:** Karolina Kuberska, Graham Martin

**Affiliations:** ^1^ Department of Public Health and Primary Care, The Healthcare Improvement Studies Institute University of Cambridge Cambridge UK

**Keywords:** cognitive impairment, dementia, hospital care, identification, nursing, patient care, person‐centred care, techno‐optimism, UK, visual identifiers

## Abstract

**Aims:**

To examine practical, ethical, and organisational implications of the use of a key technology deployed in the care of hospitalised people with dementia—visual identifiers—through a comparative analysis with parallel interventions in other spheres of healthcare and social activity.

**Design:**

Discursive paper.

**Methods:**

We contrast visual identification systems used for hospitalised patients with dementia with other, ostensibly similar, systems to understand how they differ in key characteristics: what they disclose, to whom and with what intended consequence.

**Results:**

Certain distinctive features of the ways identifier systems are used to improve dementia care appear particularly consequential for their impact. Given how much is expected of such identifiers, they are likely to fail at least a proportion of patients.

**Conclusion:**

We argue that it is important to critically evaluate the interests served by visual identifiers, identifying the dimensions of quality they can enhance and those that may be negatively impacted.

**Implications for the Profession and/or Patient Care:**

Visual identifiers for people with dementia can contribute to the ‘taskification’ of nursing care, implying that achieving person‐centred care is a matter of following defined protocols rather than an emergent, relational, time‐consuming process. Staff may end up prioritising risk avoidance and hospital routines (tasks that are measurable and auditable) rather than embracing the unpredictability of developing relationships with patients.

**Impact:**

Visual identifiers are a part of well‐established strategies to improve hospital care for those with cognitive impairment. While these identifiers aim to prompt healthcare professionals to deliver individually tailored care, research suggests that they are unable to consistently ensure the desirable quality of care. Understanding influences on how they are deployed can help reshape the expectations placed on such low‐tech interventions and inform more reflective use.

**Patient or Public Contribution:**

Patients and public were not directly involved in the development of this discursive paper.

## 1 Introduction

As populations have aged in high‐income countries such as the United Kingdom (UK), the prevalence of health conditions associated with ageing, including neuro‐degenerative illnesses such as dementia, has risen. Older people form a significant proportion of hospitalised patients: approximately 44% of inpatients in England were aged 65 or over in 2022/23, with those aged 85 or over constituting around 10% (NHS England Digital 2023). Both the numbers of older patients and the prevalence of comorbidities in this group pose challenges to the healthcare system. It is estimated, for example, that people with dementia occupy approximately a quarter of hospital beds and tend to stay in hospital twice as long as patients over the age of 65 without cognitive impairment (Murdoch and Stewart 2024). As the number of people diagnosed with dementia in the UK continues to increase (Wittenberg et al. 2019), so will the need for resources to ensure high quality‐care for this population.

Hospitals have therefore developed innovative ways of managing the care and attending to the particular needs of older people with dementia. One of the most well‐known approaches is the use of ‘visual identifiers’: small, usually subtle tokens, such as stickers placed on patient notes, special wristbands, or symbols placed at the bedside, to communicate to those familiar with their language that a patient is cognitively impaired, and invite them to adapt their approach to care accordingly. This seemingly simple intervention to try to improve care has become widely adopted, assisted in part in the UK by the recommendation by the Royal College of Psychiatrists (2019) in its National Audit of Dementia that hospitals implement such a system. While this paper focuses on the complexities of the UK context, visual identifier systems for people with dementia are also used in other countries, most notably Australia and the USA, where similar issues are likely to arise (Alzheimer's Australia 2012; Feil 2016; Hines et al. 2009).

Symbols of this kind are not unique to dementia care. Within healthcare, identifiers such as badges and stickers are frequently used to serve as reminders to busy staff, in complex care environments, of particular patient needs. Beyond healthcare, symbols such as the ‘hidden disabilities sunflower’—a ‘discreet sign that the wearer has a hidden disability’ (Hidden Disabilities Sunflower Scheme 2023)—have grown in popularity and recognition as a way of helping people to adjust their behaviour to better meet the needs of people with autism, and with other forms of neurodivergence and invisible disabilities. Yet for all their apparent potential in improving care quality and helping staff to manage their work in increasingly demanding situations, the use of visual identifiers of this sort in the UK has been subject to extensive critique (Featherstone et al. 2020; Kuberska et al. 2022; Macdonald et al. 2023; Sutton et al. 2023; Armstrong et al. 2024).

On the surface, and given the importance of ethical considerations—after all, matters of privacy, stigmatisation, and consent are important—it may seem that ethics is well placed to address most concerns. In this paper, however, we complicate the problem by approaching visual identification systems for hospitalised people with dementia as a palimpsest of overlapping, intersecting, and even conflicting issues, including but not limited to ethical ones. We argue that framing concerns about identifiers for patients with dementia primarily as a matter of ethics is a shortcut and a compromise. By directing our attention to one specific dimension of a much more complex phenomenon, the ethical lens risks obscuring the complex networks of relationships and preconceptions in which the provision of care for people living with dementia is embedded—including the day‐to‐day realities of care in wards that are often overburdened and understaffed, as well as broader influences such as the stigmatising potential of a diagnosis of dementia, enacted through stereotypes and assumptions about the condition.

Our paper proceeds as follows. First, we describe the principles and practices of visual identification for hospital inpatients with dementia in more detail, including the philosophies of care that underpin this approach, the forms that visual identifiers take in practice, and existing evidence on their impact, for better or worse, on quality of care. We summarise the main critiques of the use of visual identifiers in dementia and the evidence that suggests that they often appear to work against, rather than support, care that is more holistic, person‐centred, and sensitive to individual needs. With a view to explaining this paradox, we then seek to expand the scope of existing analyses by comparing visual identifiers for inpatients with dementia with other systems of symbols used to identify particular social needs and inform the responses of others. By drawing out points of similarity and points of divergence between these different systems, we suggest specific features of the ways identifier systems are used to improve dementia care that may be particularly consequential for their impact. A more relational understanding of what dementia identifiers do (and whom they do it for), we suggest, offers a better basis for defining the conditions in which their impact might be more negative and more positive, beyond the properties and potentials of the identifier in itself.

## 2 Background: Putting Person‐Centred Care Into Practice? Visual Identifiers as a Route to Improving Care Quality

People with dementia are usually hospitalised due to a health condition unrelated to their cognitive impairment (e.g., falls, tests, planned procedures); however, their additional needs may complicate the care they receive at hospital, potentially resulting in worse outcomes. Once there, they are more likely to experience longer inpatient stays (Möllers et al. 2018; Fogg et al. 2019). In addition to administering primary treatment, clinical staff need to address these patients' dementia‐related needs by assisting, for example, with meals, with using the toilet or with communication. This may increase the workload of already busy clinical staff, who may lack comprehensive dementia care training (Bray et al. 2015; Houghton et al. 2016; Mallon et al. 2019; Royal College of Psychiatrists 2023). Perhaps partly in consequence, patients with dementia in hospitals are at a higher risk of adverse events following admission (Möllers et al. 2018; Fogg et al. 2019; Dewing and Dijk 2016), including delirium, falls, procedure‐related complications, transfer to a higher level of care, psychological distress and unmet needs such as treatment of pain or relief of thirst (Phelan et al. 2015).

Hospitalisation presents risks to all patients—for example, the deterioration associated with prolonged stays, colloquially known as ‘pyjama paralysis’ (Oliver 2017). People with dementia, though, have a higher chance of a loss of independent functioning during a hospital stay, which in turn increases the chance of being transferred to a care home (Fogg et al. 2019; Hartley et al. 2017). Addressing the holistic needs of an individual, including their social and psychological wellbeing as well as the specific clinical need behind their hospital admission, has become a priority in recent decades, with a view both to reducing the risk of these harms and to improving the experience of patients more broadly. A prominent approach of this kind is the philosophy of ‘person‐centred care’ (Abbott et al. 2022), which aims to promote wellbeing and minimise distress by meeting the fundamental physical, psychological, and social needs of people receiving healthcare. Given the additional risks they face, achieving person‐centred care for hospitalised people with dementia is particularly important. Key features of a person‐centred approach to caring for people with dementia might include taking time to get to know them, adapting the physical care environment, and involving family and carers (Abbott et al. 2022), among others. Above all, the idea is to treat the person as a unique human individual, and to avoid reducing them to their diagnosis.

Translating the principles of person‐centred care into practice often involves the codification of the specific steps that need to be taken to achieve it, such as activities on the part of staff or the use of technologies, simple or complex. In relation to ‘information sharing’, Abbott et al. call on practitioners to

make space to document psychological wellbeing and/or distress. Use simple systems to identify whether someone has dementia: this can help remind everyone to take more time with care. Share personal likes and dislikes, and individual behaviours (preferred name, family situation). (2022, 4)

These ‘simple systems’ have taken the form of a wide variety of visual identification approaches. Signs or symbols have been used to indicate a hospitalised patient's diagnosis of dementia for a couple of decades (Hines et al. 2009). Identifiers currently in use take the form of stickers (e.g., with butterfly or forget‐me‐not symbols) placed on patient notes and/or over the bed, symbols on electronic boards or whiteboards, special‐coloured (often blue) wristbands, flower‐shaped cut‐outs in standard hospital wristbands, and magnets depicting purple angels or forget‐me‐not flowers placed on ward boards (Featherstone et al. 2020; Kuberska et al. 2022; Sutton et al. 2023). The purpose of such identifiers is to signal to healthcare staff that a patient may have additional care needs related to their diagnosis of dementia, so that staff can tailor the way they interact with the patient and fulfil the promise of person‐centred care. In addition to registering the diagnosis of dementia in patient records, some hospitals use additional patient profile documents, such as the Alzheimer's Society's ‘This is me’ booklet, kept by the patient's bed, with key information about the person, their preferences, and habits noted by a family member, friend or other informal carer (sometimes in cooperation with the person with dementia) to help hospital staff recognise that person as an individual (Kuberska et al. 2022; Sutton et al. 2023). The implied simplicity of those systems may, however, be superficial. Although many visual identifications systems are technologically uncomplicated, their use and presence in the hospital environment are by no means simple, as shown by various studies (Armstrong et al. 2024; Featherstone et al. 2020; Kuberska et al. 2022; Macdonald et al. 2023; Sutton et al. 2023).

## 3 Overview of Issues: Visual Identifiers in Practice—Empirical Evidence on Their Impacts

Uptake of visual identification systems of this kind is now widespread in the UK, aided both by the development and promotion of such schemes by a variety of organisations outside the healthcare system such as charities and campaigning groups, and by ‘top‐down’ recommendations that hospitals adopt one, emanating for example from professional associations. The National Audit of Dementia, run annually by the Royal College of Psychiatrists, asks whether participating hospitals follow its recommendation to implement a formal system for identifying patients with dementia. In both rounds 4 and 5 of the audit, 97% of organisations indicated that they had such a system in place (Royal College of Psychiatrists 2019, 2023). While their status as a recommended practice, and one where compliance is measured and regularly publicly reported, may explain the spread of visual identifier systems for inpatients with dementia, there also appears to be broad enthusiasm among healthcare practitioners and organisations. Glowing reports of locally implemented initiatives are easily found on hospital websites, with headlines such as ‘Patient bands are worth their weight in gold for improving care’ (South Tyneside and Sunderland NHS Foundation Trust 2019).

With their popularity has come significant academic scrutiny. Empirical study of the use of visual identifier schemes has identified their flaws and unintended consequences (Armstrong et al. 2024; Boddington et al. 2024; Featherstone et al. 2020; Featherstone and Northcott 2020; Kuberska et al. 2022; Sutton et al. 2023). Different types of visual identifiers for patients with dementia have different (partially overlapping) benefits and risks, and many UK hospitals use more than one system for such patients (Featherstone et al. 2020; Kuberska et al. 2022; Royal College of Psychiatrists 2023; Sutton et al. 2023). However, even with multiple available prompts, it is not always possible for every need to be addressed at all times in all patients with dementia. One reason is the busyness of clinicians, working in sometimes understaffed wards, who need to prioritise urgent needs over less urgent ones. For instance, when patients who also have dementia walk about and leave the ward (or even the hospital), finding them will take precedence over addressing any additional needs they or other patients might have. More generally, the sheer proliferation of such symbols and the way this can (ironically) add to rather than simplify the cognitive demands on the intended recipients of the information they seek to communicate (Allen 2017; Featherstone et al. 2020; NPSA 2007) creates new risks for quality of care. In wards with high staff turnover or with a considerable proportion of bank staff, recognition and comprehension of identifiers may be partial, limiting the extent to which they can fulfil their intended function. Concerns have also been raised regarding patient privacy, quality of consent and the type of information revealed (Boddington et al. 2024; Brigden et al. 2020, 2023; Featherstone et al. 2020; Kuberska et al. 2022; Sutton et al. 2023). In consequence, visual identifiers may fall well short of their promise for person‐centred care.

There have been notable efforts to address concerns of this kind. A study aiming to develop design principles for visual identification systems for inpatients with dementia, for example, foregrounded the importance of a well‐functioning care environment. Such an environment would be characterised by attention to multiple key features in parallel, from providing appropriate training to staff to ensure that the identifier is consistently recognised correctly, through securing reliable access to relevant information about patients (both in paper documents and on the electronic patient record), to involving family and carers in implementation (Macdonald et al. 2023). Similarly, an analysis of the ethical and legal concerns around using visual identifiers for hospitalised people with dementia suggested a set of seven principles, familiar from the field of bioethics, for using this type of intervention in the spirit of person‐centred care (Brigden et al. 2023): dignity, compassion, autonomy, justice, holistic care, confidentiality and privacy, and beneficence and non‐maleficence. Since these principles may conflict in practice, Brigden et al. (2023) emphasise that achieving person‐centred care is dependent on making situational judgements about their relative importance that account as far as possible for the particularities of the situation and the preferences and interests of the patient.

## 4 Findings: What's Behind the Shortcomings of Visual Identifiers in Dementia? A Comparative Analysis

Efforts of this kind to address the shortcomings of visual identification systems and optimise their implementation are undoubtedly valuable, especially given the prevalence of such systems. However, they have tended to focus, first, on addressing the limitations intrinsic to an identification system (e.g., how the issue of inadvertent disclosure of private information might be managed) and, second, on improving its compatibility with a complex system of care that can compromise its efficacy. We argue that, while important, these issues are not the only ones that give rise to the situation described in the section above, in which systems implemented with a view to realising the principles of person‐centred care appear to do exactly the opposite.

To elaborate this argument and identify the other influences that might be behind this situation, we next present a comparative analysis of various other visual identification systems in use within and beyond healthcare. Examining apparently similar interventions side by side allows us to parse the issues that might be associated with visual identification as a technology in general, the issues that relate to the way it is used in dementia care specifically, and issues whose causes may lie less with identifiers themselves and more in wider social, institutional and cultural influences. Acknowledging that there are many more systems that are possible to discuss here, we focus on a selection of systems with features that partially overlap with the characteristics of visual identifiers for inpatients with dementia. We discuss how they align and diverge in terms of consent, recognisability, what the identifier is supposed to signal, and the social perception of the condition or trait associated with the identifier. Our three comparator identifiers are red hospital wristbands, traffic light hats for newborn babies and hidden disability sunflower lanyards.

### 4.1 Red Hospital Wristbands

Red hospital wristbands, a variation on standard white hospital identification wristbands, are perhaps the least conspicuous of all the visual identifiers used in healthcare. They are used to identify patients with a known risk, such as a medication allergy (NPSA 2007). In the UK, following a 2005 report pointing out safety issues due to missing wristbands, the National Patient Safety Agency (NPSA) called for standardisation of all patient wristbands and information placed on them, recommending the use of only white patient identity bands and red ones for patients with known risks (NPSA 2007). In hospitals where red patient wristbands are used, staff are expected to double‐check the specific risk the wristband indicates. In other words, the identifier demands a very narrow and specific action on the part of a specific intended actor, and one that is clearly critical to the safety and wellbeing of the person wearing the wristband. Typically, consent is not explicitly sought for the use of the wristband for hospital patients, even when the wristband may reveal medical information that would normally be confidential, such as the allergy in question.

The limited available literature on coloured wristbands of this kind identifies benefits of these identifiers for patients in the form of improved safety and error reduction (Sevdalis et al. 2009) while acknowledging problems that may arise, from patients inaccurately reporting their own allergies (Clark et al. 2018) to inconsistent use (Clark et al. 2018; Nitro et al. 2021). It is interesting to note that patient privacy (unlike risk of error) is not a prominent theme in the academic literature on these wristbands. Though private, the information they reveal (such as risk of allergy) is not obviously sensitive or stigmatising, and so concerns of this kind are perhaps more easily dismissed. Red hospital wristbands signal significant and avoidable risks, and the advantages of making those risks prominent to staff outweigh, it seems, the possibility of the identifier revealing specific information about the patient without their consent, to the extent that any potential concerns about privacy (of the kind very prominent in discussions of dementia identifiers) are simply not part of the equation.

### 4.2 Traffic Light Hats for Newborns

In some hospitals, newborn babies are classified with a visual identification system. This system consists of traffic light‐coloured hats put on babies in neonatal units to indicate the level of support they need. A green hat indicates that the newborn requires routine care and checkups, an amber hat signals that some extra support is needed (for instance following a complicated labour), and a red hat indicates that the baby requires more frequent observations (Pengelly 2019; PAT 2018). The traffic light hat system aims to assist hospital staff in their care for newborn babies—premature and full‐term—not only through their direct physiological impact in helping to maintain babies' body temperature at the right level, but also by serving as visual reminders for staff to check on the newborns with frequency appropriate to their condition (Hutchinson 2019; PAT 2018; Pengelly 2019). This visual identification system appears to be popular—as informally evidenced by the multitude of glowing articles on the internet about yet another NHS maternity ward adopting it (PAT 2018; Pengelly 2019; Hutchinson 2019). Some dissenting opinion can also be found, for example on midwifery‐themed blogs. This relates less to the identifying function of the hats, and more to their material impacts: for example, questions of whether newborns need hats for thermoregulation at all, whether donated hats are newborn‐safe, and whether these babies would be better off receiving skin‐to‐skin care than relying on a hat (Analytical Armadillo IBCLC 2019; Juno Midwives 2023).

Like dementia identifiers, then, these traffic light hats for infants appear to be popular with healthcare staff. Similar to allergy wristbands, they are subtle: other than those who have been specifically briefed, observers are unlikely to infer information about an infant's vulnerability from an array of hats in primary colours. Like allergy wristbands (and in contrast to dementia identifiers), there appear to be few concerns about issues such as disclosure of personal information. Nonetheless, despite the widespread enthusiasm, adopting traffic light hats for newborns is not entirely unproblematic. Clearly newborns themselves cannot consent to wearing the hats, but neither is consent routinely sought from the parents, even though the visual identifier reveals private information about the patient's condition to those who can read the system. Hospitals' policies around using this kind of identifier might rely on implied consent, under the assumption that the benefits (for babies, staff and even for parents who might find the hats reassuring) significantly outweigh the risks (e.g., of parents who may not want to be reminded that their baby is poorly). Additionally, some maternity wards use the hats only briefly, for the first 24 h following birth (Hutchinson 2019; PAT 2018), perhaps leading to a view that an informed consent process would be disproportionate.

Perhaps the most important difference between newborn hats and dementia identifiers in explaining the rather divergent responses with regard to privacy and disclosure, however, is that the information revealed by the hats is limited to the fact that some babies may simply need more frequent observations than others. They do not reveal the underlying reasons for the need for more frequent observations. In other words, what the identifier signals is unlikely to be even potentially stigmatising for the baby (or the parents), and nor is it something that is likely to have other adverse impacts on them. What this comparison suggests, then, is that what matters is the *kind* of information that a visual identifier may reveal (such as a specific diagnosis)—rather than the fact that it can reveal information per se—as well as to whom it is likely to reveal this information.

### 4.3 Hidden Disability Sunflower Lanyard

An interesting example of a visual identifier used beyond healthcare settings is the hidden disability sunflower lanyard. This now‐international scheme involves green lanyards with images of sunflowers that can clip onto cards with information, specific or general, about the person's hidden disability (Hidden Disabilities Sunflower Scheme 2021b). Lanyards can be worn by anyone who has a hidden disability and who could benefit from wearing a lanyard (Hidden Disabilities Sunflower Scheme 2021). They are distributed for free by organisations that are members of the scheme or can be purchased from the online store of the charity that organises the scheme, where individuals are also able to buy personalised hidden disability cards featuring their photo and a more detailed description of their hidden disability. The lanyards are to be used in public places where a person with a hidden disability might benefit from assistance, such as banks, railway stations, airports, cinemas and supermarkets. Importantly, wearing these lanyards is voluntary and an individual chooses whether or not to wear the identifier at any given time or place.

Because of public awareness campaigns, as well as training of staff in participating organisations, the lanyard is widely recognised, as evidenced by over 1800 locations in the UK alone where the scheme has been implemented (Hidden Disabilities Sunflower Scheme 2021c). The scheme's broad appeal is anchored in choices of symbols and colours evoking positive associations (sunflowers against a vibrant green background), multiple positive awareness campaigns, official training courses for member institutions and businesses, relying on individuals' self‐determination regarding whether they would like to wear it, and the representational capacity of the sunflower lanyards to make hidden disabilities visible. These last two features of the scheme may be particularly empowering to those with non‐visible disabilities, positioning the sunflower lanyard as a visual identifier that can challenge—rather than perpetuate—social stereotypes associated with hidden disabilities (Fisher 2023).

At the same time, the scheme's usefulness for people with certain types of invisible disabilities, such as autism, has been criticised as tokenistic (MacLennan et al. 2023). Some users report not receiving the help they require to navigate public spaces, even where staff were supposed to have received training. Others point to staff making unfounded assumptions about their hidden disability (Fisher 2023; MacLennan et al. 2023).

Unlike dementia identifiers, then, there are no questions about the ethics of disclosure. Whether to wear a sunflower is a matter of self‐determination by an individual with the capacity to weigh the pros and cons of the decision. While public understanding of hidden disabilities, such as various forms of neurodivergence, has increased in recent decades, there remains some evidence of stigmatisation, but this is a risk for the individual to consider against the potential benefits of wearing one. However, in common with dementia identifiers, the hidden disability sunflower lanyard produces a tension between its function in signalling that the person wearing it may possibly need help, and the fact that it cannot specify the appropriate form of assistance in any given situation. Like visual identifiers for hospitalised people with dementia, what is indicated by a sunflower is insufficiently specific to be addressed correctly at all times. Indeed, in some ways their function is even more complicated than that of identifiers used for people with dementia in hospital, given that they might signal different needs for different people in different contexts: one individual's reason for wearing it may differ from another's, and the same person might hope for different responses to the sunflower lanyard in, say, a railway station and a bank.

## 5 Discussion

The selective comparison of different identification systems is instructive, we suggest, in a number of ways. First, inadvertent disclosure of confidential information is a risk common to several of the identifiers—including situations where the subject of that confidential information has not consented to, or perhaps even been informed of, this risk. However, the consequences of disclosure are not equally grave across all identifiers—to the extent that academic critique of some of the indicators does not even consider it worthy of comment. This is not, it seems, because of the narrowly ethical question of whether informed consent has been sought and given (only in the case of sunflower lanyards is this clearly consistently the case) but because of the gravity of the consequences of disclosure. In the case of traffic light hats for newborn babies, those able to read the identifier beyond the intended audience are informed only of the need for more attentive monitoring from care‐giving staff, not of the child's specific medical circumstances. Red wristbands offer more specific information, of the kind that could be considered sensitive and personal. Dementia identifiers, however, have the potential to expose a diagnosis that remains stigmatised and which both the individual and their family might wish to remain private. An allergy, a higher risk of complication for a newborn, and even a hidden disability are things that a person *has*: a diagnosis of dementia is often seen as changing who a person is (see e.g., Cruise and Lashewicz 2022; Pelters 2024). The social consequences of disclosure for the individual and those around them could be noticeable and may not be welcome.

Second, conversely, the information that the identifier is intended to impart is quite different across the four examples. Red wristbands in particular seek to communicate something that is straightforward but highly consequential—where miscommunication of a simple but vital piece of information could have profound negative consequences. The risk–benefit ratio in this case appears clear and again, the simplicity of the equation means that the ethical question of consent seems not to enter the academic discourse, which is concerned only with questions of effectiveness.

Third, and closely related, what is to be done by the receiver of the information varies markedly across the identifiers. For red wristbands, the implication is relatively straightforward: avoid the use of medicines that contain the relevant allergens. (The ease of putting this instruction into practice may be affected by the quality of wider socio‐technical systems in the hospital, of course, but the instruction at least is unambiguous.) For traffic light hats, again, the person receiving the information has a relatively simple task to pursue: choosing the right newborn monitoring regime to follow. For sunflower lanyards and dementia identifiers, however, the right course of action is much more ambiguous. In both cases, a simple message (about the characteristics or needs of the individual) begets much more difficult questions: So what? How should I best respond? What does a caring, sensitive, or person‐centred response look like right now—for this individual, in this situation, given the specific objectives of this interaction? These, of course, are matters of judgement.

Fourth, in the case of both sunflower lanyards and dementia identifiers, this judgement is often supported by the provision of additional information of the kind that is not needed in the interpretation of allergy wristbands or newborn hats. This includes cards containing personalised information that the wearers of sunflower lanyards may choose to carry, or more detailed information about a patient with dementia, such as their personal history, likes and dislikes, and details of their family and friends. However, with the addition of this information and the expectation that it be used by staff providing care, the simplicity and intuitive appeal of the identifier are lost. Moreover, these supplementary materials may not always be supplied in the first place, they may easily become detached from the identifier, and even where they are available, they may not always be consulted—whether by a passer‐by in a railway station or a busy nurse on a ward (Challis et al. 2022; Featherstone et al. 2020; Kuberska et al. 2022; Sutton et al. 2023).

In short, then, neither ethical issues relating to informed consent, capacity, privacy and disclosure, nor the challenges of integrating dementia identifiers into complex systems, can fully account for the issues associated with their use. Rather, our analysis suggests two other vital issues: the nature of the challenges faced by people with dementia and those close to them during a hospital admission; and the extent to which simple solutions like identifiers can reasonably be expected to mitigate those challenges.

## 6 Conclusion: Visual Identifiers and the Pursuit of High‐Quality Care

As others have found (Featherstone et al. 2020; Featherstone and Northcott 2020), the upshot of the issues in the use of visual identifiers for hospitalised people with dementia is that, in at least some circumstances, they result in exactly the opposite of what they intend. A combination of the scale of potential negative impact of the disclosure (arising from the way in which a dementia diagnosis is culturally constructed), the ambiguity of the information transmitted, and the difficulty of identifying the appropriate response means that when introduced into busy environments with overburdened staff, these identifiers become at best a blunt tool for achieving person‐centred care. At worst, they risk leading to harm by reinforcing the reduction of a person to their condition. However inadvertently, identifiers can contribute to the ‘taskification’ of care, implying that achieving person‐centred care is a matter of following defined protocols rather than an emergent, relational, time‐consuming process. In busy wards, staff may end up prioritising risk avoidance and hospital routines (i.e., tasks that are measurable and auditable) rather than embracing the unpredictability of developing relationships with every patient (Allen 2016, 2017; Leary and Punshon 2024).

Thus, the challenges of dementia identifiers are not reducible solely to ethical considerations, as important as these are. As our comparison shows, issues of consent, privacy and discretion would not be so important were it not for the impact of dominant assumptions and (mis)understandings about dementia at the societal level. These relate not only to the potential discrimination that may follow from inadvertent disclosure, but also to the risks of poor‐quality care that can follow even when the identifier communicates the correct information to the intended audience. For example, hospital staff may be insufficiently trained, insufficiently experienced or simply too busy or tired to consistently provide optimal care to hospitalised people with dementia, irrespective of whether they are able to see the patient identifier.

In consequence, this seemingly simple and intuitive intervention can easily malfunction. Efforts to mitigate this risk, such as booklets providing additional information about the person with dementia, are easily lost or disregarded; moreover, they compromise the main appeal of identifiers: their ostensible simplicity (Boddington et al. 2024; Brigden et al. 2023; Sutton et al. 2023). Here, one of the key drivers of use of visual identifiers for dementia—their prominence in a national audit which publishes organisations' uptake of such systems—may risk exacerbating their unintended consequences. Monitoring use of visual identification systems in this way may encourage *adoption* over a more considered approach to *implementation* (Allen 2017). If an intervention is adopted without considering how it fits in with what is already there, unintended consequences are likely. In the case of visual identifiers for dementia, we know that some hospitals patch the gaps in coverage with additional identifier systems that add to the staff workload and crowd the individual patient signage landscape—without necessarily improving care (Featherstone et al. 2020; Kuberska et al. 2022). At the same time, the presence of identification systems for hospitalised people with dementia can be easily captured in audits, perhaps creating an impression that they work as intended.

What, then, might be a better way of getting the best out of visual identifiers for dementia, and ensuring that they contribute to care that is better for the individual patient? Our comparison suggests that reframing our expectations of what such an identifier can achieve might have some promise. Red wristbands and traffic light hats appear to be more successful—or at least less controversial—because the simplicity of their message is matched by the simplicity of the expected response. Particularly in crowded, busy and understaffed care environments, reining in the ambitions for identifiers might be an appealing option. By recognising the strengths and limitations of visual identifiers in this way, their unintended negative consequences for person‐centred care might be avoided. Instead of serving as a substitute for holism, their role could be limited to ensuring the completion of simple but vital, sometimes even safety‐critical, tasks.

This also leaves outstanding the question of exactly what role the technology of the visual identifier could continue to serve for hospitalised people with dementia specifically. Given that patients with a dementia diagnosis who are admitted to hospital will have different needs and preferences, be at different stages of the disease, differ in their lucidity, and so on, it is difficult to imagine exactly what an identifier might signal. It is, of course, important to know that a patient has dementia. Capturing information that a patient has this diagnosis can be crucial for setting up their hospital care appropriately—for example by avoiding ward transfers or allocating rooms with visual prompts for those patients with dementia whose visual impairment prevents them from seeing depth well or whose memory loss makes it difficult to remember room numbers. Achieving these objectives, however, is less about sensitising individual staff to a patient's diagnosis so that they can enhance patient‐centredness through their interpersonal interactions, and more about implementing high‐quality systems to optimise the organisation of care.

Techno‐optimism in healthcare is underpinned by the conviction that introducing a piece of technology can—and will—be a gamechanger (Wilson 2017). Although the low‐tech visual identifiers discussed in this paper are not a classic manifestation of techno‐optimism, they share several characteristics of its logic, where the success of a procedure, care service or diagnosis is ascribed to an object. In reality, this object usually works as intended (or doesn't) because of multiple contextual factors, crucially including human ones (see e.g., Allen 2016). Anthropology has long described the social lives of objects, from commodities to ritual artefacts (Appadurai 1986). Similarly, the institution of medicine constructs its objects using unspoken rules and insistence on conformity and uniformity (Good 1993), further reified by the proliferation of tick‐box procedures, repetitive mandatory training and audit trails (Allen 2017). But these are not what secures the success of a new technology, complicated or simple—and sometimes they might even subvert it.

This all points, perhaps, to a relatively limited role for visual identifiers in dementia care. Yet visual identifiers for people in hospital with dementia are, as we have seen, part of the fabric of many hospitals; given the frenetic nature of inpatient care, increasing workloads and the huge pressures on resources, they may even have come to be crucial as a shortcut for hard‐pressed healthcare staff. But in serving this function, they risk prioritising the efficiency of care over other aspects of its quality, particularly from the perspective of patients and families. Resetting the role of simple technologies in healthcare—ensuring that their role and remit do not exceed the limits of their positive capacities—may be crucial in reconciling quality, safety, patient experience and productivity.

## Author Contributions

**Karolina Kuberska, Graham Martin:** made substantial contributions to conception and design, or acquisition of data, or analysis and interpretation of data. **Karolina Kuberska, Graham Martin:** involved in drafting the manuscript or revising it critically for important intellectual content. **Karolina Kuberska, Graham Martin:** given final approval of the version to be published. Each author should have participated sufficiently in the work to take public responsibility for appropriate portions of the content. **Karolina Kuberska, Graham Martin:** agreed to be accountable for all aspects of the work in ensuring that questions related to the accuracy or integrity of any part of the work are appropriately investigated and resolved.

## Ethics Statement

This article did not require ethical approval because it is a discursive paper examining the issues around using visual identifiers for hospitalised patients with dementia.

## Conflicts of Interest

The authors declare no conflicts of interest.

## Data Availability

The authors have nothing to report.
